# A pipeline for the fully automated estimation of continuous reference intervals using real-world data

**DOI:** 10.1038/s41598-023-40561-3

**Published:** 2023-08-18

**Authors:** Tatjana Ammer, André Schützenmeister, Hans-Ulrich Prokosch, Manfred Rauh, Christopher M. Rank, Jakob Zierk

**Affiliations:** 1 Chair of Medical Informatics, Friedrich-Alexander-Universität Erlangen-Nürnberg, Erlangen, Germany; 2 Roche Diagnostics GmbH, Penzberg, Germany; 3 Department of Pediatrics and Adolescent Medicine, Universitätsklinikum Erlangen, Loschgestr. 15, 91054 Erlangen, Germany; 4 Center of Medical Information and Communication Technology, Universitätsklinikum Erlangen, Erlangen, Germany

**Keywords:** Data mining, Statistical methods, Paediatric research, Biomarkers, Diagnostic markers, Laboratory techniques and procedures

## Abstract

Reference intervals are essential for interpreting laboratory test results. Continuous reference intervals precisely capture physiological age-specific dynamics that occur throughout life, and thus have the potential to improve clinical decision-making. However, established approaches for estimating continuous reference intervals require samples from healthy individuals, and are therefore substantially restricted. Indirect methods operating on routine measurements enable the estimation of one-dimensional reference intervals, however, no automated approach exists that integrates the dependency on a continuous covariate like age. We propose an integrated pipeline for the fully automated estimation of continuous reference intervals expressed as a generalized additive model for location, scale and shape based on discrete model estimates using an indirect method (refineR). The results are free of subjective user-input, enable conversion of test results into z-scores and can be integrated into laboratory information systems. Comparison of our results to established and validated reference intervals from the CALIPER and PEDREF studies and manufacturers’ package inserts shows good agreement of reference limits, indicating that the proposed pipeline generates high-quality results. In conclusion, the developed pipeline enables the generation of high-precision percentile charts and continuous reference intervals. It represents the first parameter-less and fully automated solution for the indirect estimation of continuous reference intervals.

## Introduction

Reference intervals (RIs) are an important tool for facilitating the interpretation of numeric laboratory test results^1,2^. By definition, RIs describe the interval where most likely non-pathological results should reside. They are usually determined by defining the central 95% range of the distribution of samples from ‘apparently healthy subjects’ enrolled into a prospective study with defined inclusion and exclusion criteria. RIs, or more precisely the test results for biomarkers, can be influenced by different covariates, like sex, region, or ethnicity. Many biomarkers are also highly dependent on the covariate age and show extensive dynamics during physiological development, especially during infancy, puberty, and in the elderly^3–5^.

One established approach to represent age-dependent dynamics is to define discrete age-groups and determine age-group specific RIs. However, these discrete groups can only capture the underlying physiological dynamics up to a certain degree and lead to unnatural, discontinuous transitions between age groups (i.e. step-function), opposed to the underlying smooth transition^6–9^. To circumvent this problem, continuous RIs have been proposed^5,9–11^. One example for such continuous, age-dependent curves are the WHO growth charts for newborns and young children^12^.

However, to establish such RI curves, and in turn to adequately model the pronounced age-dependent dynamics, a vast amount of samples is needed. Obtaining these large numbers of samples is often impossible with population-based techniques requiring sample collection studies, due to ethical, practical, and logistical challenges^5,11^.

Indirect methods, operating on routine measurements (real-world data, RWD) can help mitigating this problem, as the data is readily available in laboratory information systems. Different approaches for estimating continuous RIs using either direct or indirect methods exist, e.g. using penalized splines or fractional polynomials in combination with manual tuning of the smoothing parameters^13–17^. However, no indirect method is available that integrates the continuous change of covariates, such as age and that does not require manual tuning^11^. Hepp et al. have recently developed a one-step solution to estimate continuous RIs using routine measurements, however the strong model assumptions (i.e. Gaussian distribution of test results) limit its clinical application^18,19^.

Here, we present a novel, integrative and automated pipeline employing routine measurements (RWD), an indirect method (refineR), and a sophisticated statistical approach for the estimation of smooth curves (Generalized Additive Models for Location, Scale, and Shape, GAMLSS) to create continuous RIs and percentile charts. All components of the pipeline are available as open-source and to execute it, an R-script^20^ with example code is provided in the Supplemental Material.

To showcase the application of the pipeline, we estimate continuous RIs with CIs using pediatric routine measurements of three biomarkers known for their extensive pediatric dynamics and compare the results to established RIs from the CALIPER studies^21,22^, the PEDREF^13,14^ project as well as from the package inserts provided by the manufacturers^23–27^.

## Methods

### Overview of automated pipeline

**Figure 1:**
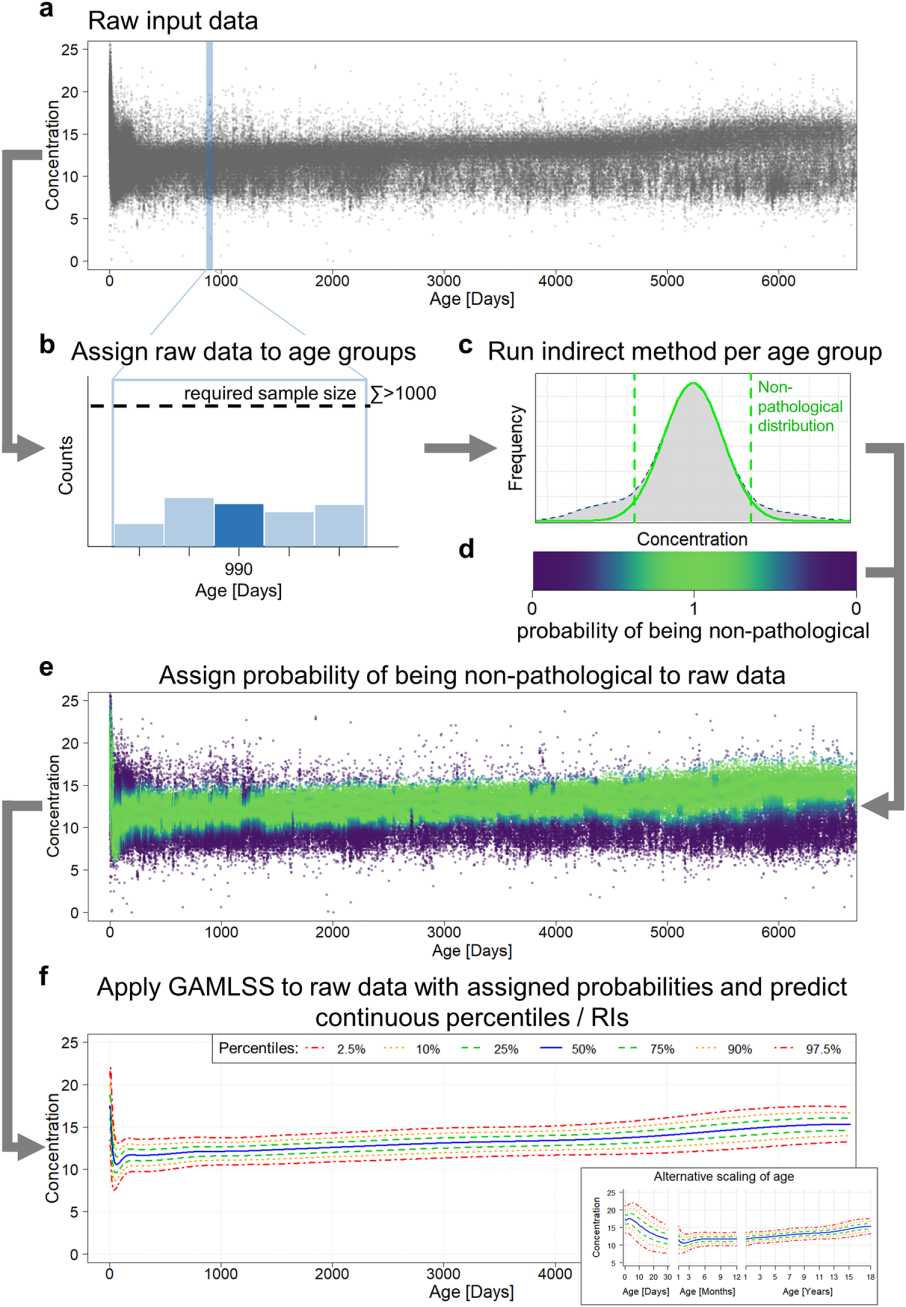
Flowchart of the developed pipeline for the estimation of continuous reference intervals from routine data. Based on the raw input data (**a**), overlapping age groups with at least N = 1000 samples are defined (**b**). An indirect method (here refineR) is applied to each defined age group to estimate a model describing the non-pathological distribution (**c**). This model is then used to compute a probability of being non-pathological along the concentration range (Eq. (1)), i.e. that a data point with that concentration originated from the non-pathological distribution (**d**). The light green colored area represents a probability of 1 (most likely non-pathological), and the dark blue colored region a probability of 0 (most likely pathological) (**d**). These estimated weights are then assigned to each data point (**e**). Following that, a statistical method to estimate smooth curves (here GAMLSS) is applied to the weighted input data set. The resulting parametric model enables the derivation of continuous reference intervals and percentile charts (**f**). To interpret and analyze pediatric data in more detail, percentile charts can also be presented with alternative scaling of the covariate age (**f** inset).

Our developed pipeline leverages routine measurements and sophisticated statistical tools to estimate continuous RIs. For this purpose, we utilize a state-of-the-art indirect method for RI estimation (refineR^28^) in combination with a statistical method to estimate smooth curves (GAMLSS^29^). The pipeline is highly modular and operates in a sequential procedure. It consists of four main steps (Fig. 1):

First, the raw input data (Fig. 1a) are assigned to overlapping age groups with a high temporal resolution (1) (Fig. 1b). For each of these groups, an indirect method (here refineR^28^) is applied to estimate a model describing the non-pathological distribution (2) (Fig. 1c). These estimated models are then used to assign a weight to each raw data point describing the probability of being non-pathological, i.e. originating from the non-pathological distribution (3) (Fig. 1d, e). Following that, a statistical method to estimate parametric, smooth curves (here GAMLSS^29^) is applied to the raw input data while taking the assigned probabilities into account. This parametric model then enables the derivation of age-specific percentiles representing estimates of continuous RIs and percentile charts (4) (Fig. 1f). In addition, the model allows for conversion of test results into z-scores.

A more detailed description of the individual steps is given in the following sub-sections.

### Definition of age groups with high temporal resolution

The first step in the pipeline is to assign the data to overlapping age groups. In order to model the dynamics observed in early infancy and puberty appropriately, we define age groups with high temporal resolution (Fig. 1b). After birth, age groups are constructed for each day of life. To ensure a sufficient amount of data for the application of the indirect method, we expand each group equally to the ‘left’ and the ‘right’ until we reach a minimum of N = 1000 samples per group. For this minimum sample size, refineR was previously shown to achieve robust results for fractions of pathological samples  ≤ 20%^30^. If there are multiple samples per subject in one age group, only the measurement with the most ‘central’ age is used and the other samples are removed in this group to avoid intra-patient correlation effects^31^. To reduce computation time, the width of the groups is linearly expanded with increasing age. We employ a 1% increase of width based on the age in days, as it is assumed that physiological dynamics are most pronounced and rapid in the first days and months of life^5,14^ (e.g. for a group with age at 1000 days, the expanded age group would cover the range 995–1005 days). This 1% increase of age range reduces the total number of age groups from 6570 to 479 in our case.

### Estimation of age-group specific non-pathological models using indirect method

For each of the age groups defined in step 1, an indirect method is applied to estimate age-group specific RIs and models describing the underlying non-pathological distribution (Fig. 1c). Here, we apply the refineR algorithm^28^ (refineR v1.5.1) to the different groups.

The refineR algorithm, as all indirect methods, operates on routine measurements and assumes that these consist of a mixture of pathological and non-pathological samples with the latter being in the majority. Further, it is assumed that the distribution of the non-pathological samples can be modeled with a (1- or 2-parameter) Box-Cox transformed normal distribution. After a pre-processing step to determine the central concentration region, a multi-level grid search is carried out to find the model that best describes the histogram of the raw routine measurements. refineR was recently described and evaluated in detail^28,30^ and could demonstrate convincing performance for sample sizes as small as N = 1000 with a pathological fraction of up to 20% as well as for different distribution types, reaching from normal over skewed to heavily skewed distributions^30^. The algorithm is provided as an open-source R-package on CRAN (https://cran.r-project.org/package=refineR).

### Computation of probabilities of being non-pathological

In a next step, the estimated models describing the non-pathological distribution for each age group are utilized to compute and assign a weight to each raw data point reflecting its probability of being non-pathological.

For each age group and thus each parametric model, the weights are computed as the ratio of densities of the estimated model and the total distribution of the raw data within this group (Eq. (1), Fig. 1d).

1 $$P\left(conc, a\right)=\mathrm{min}\left(1,{\text{max}}\left(0,\frac{{D}_{np}\left(conc, a\right)}{{D}_{total}\left(conc, a\right)}\right)\right)$$

with *conc* = concentration, *a* = age group, *D*_*np*_ = density of non-pathological distribution, *D*_*total*_ = density of total distribution of raw input data. To increase robustness for the estimation of *P*, slight Gaussian smoothing is applied to the concentration-dependent ratio.

A probability *P* of 1 denotes that samples are assumed to originate from the non-pathological distribution (e.g. located within peak region) (Fig. 1d light green region), while a probability of 0 means that samples are most likely pathological (e.g. located in outer regions) (Fig. 1d dark blue region). Between these two extreme points, there is a transition region where continuous probabilities between 0 and 1 are assigned to the samples (Fig. 1d light blue region). Overall, the samples within the peak region contribute more to the GAMLSS estimation than those at the outer regions (Fig. 1e).

To account for the fact that one subject can contribute with multiple samples to the whole dataset leading to potential bias caused by intra-subject correlation in distinct age-classes, the data points are weighted down if the subject is represented multiple times as described in Eq. (2):

2 $$P\_corr\left(subject, conc, a\right)=\frac{P(conc,a)}{{N}_{subject}}$$

with *N*_*subject*_ = number of samples for a subject in the whole dataset, and *P(conc, a)* as described in Eq. (1).

As the age groups are occasionally expanded and thus overlap, the weight within each age group is assigned only to the ‘central age’. Hence, exactly one weight is set to each data point and is used in the following step to indicate the probability of individual samples being non-pathological (Fig. 1e).

### Estimation of parametric, smooth curves using GAMLSS

Having assigned the weight or probability of being non-pathological to each data point (Fig. 1e), the next step is to estimate a parametric model for the continuous RIs. Here, we utilize the generalized additive models for location, scale, and shape (GAMLSS)^29^.

Specifically, we apply the LMS (lambda-mu-sigma) method with extensions, with the basic assumption that the response variable follows a distribution that depends on the covariate of interest, here the age of the subject. Similar to the refineR model assumptions, the LMS method, or also known as Box–Cox Cole and Green distribution, fits three parameters: skewness (lambda), location (mu), and scale (sigma)^32^. In order to allow for more flexibility in model fitting, additional distribution families, which can be seen as extensions to the LMS method, are tested. Namely, the Box-Cox power exponential and the Box-Cox t-distribution. Both allow for fitting a forth parameter that models the kurtosis of the distribution^33^. These different distribution models were recently shown to achieve good performance in estimating continuous RIs for non-pathological datasets^34^. The best model out of these different distribution families is then selected based on the Bayesian Information Criterion (BIC).

The GAMLSS framework (https://cran.r-project.org/package=gamlss) is utilized to estimate a parametric model for the continuous RI curves. It results in a single model representing the age-dependent RI curves.

### Derivation of continuous RIs and percentile curves

After computing the parametric model, it can be used to estimate percentile charts and thus continuous RIs over the range of the covariate, here from birth to adulthood (Fig. 1f). Additionally, the parametric model allows for conversion of test results into z-scores. The 2.5th and 97.5th percentiles, i.e. the ‘typical’ reference limits, would correspond to a z-score of approximately −1.96 and +1.96. Making use of z-scores corresponds to normalizing RIs and laboratory test results and thus facilitates the interpretation of the latter^13,35^.

### Estimation of confidence intervals

In order to provide CIs for the estimated continuous RIs we use bootstrapping. Thus, we randomly sample from the input dataset with replacement and use the randomly sampled data set as input for the pipeline. The pipeline is then executed 100 times resulting in 100 parametric GAMLSS models. These are then used to predict the different percentiles for each day of life and the CIs are computed as the central 95% region of the estimated percentile points. We found that the estimated CIs can occasionally be strongly asymmetric causing the original model estimated on the whole data set to lie outside of the central 95% region. In such situations, the CI was expanded to include the estimate of the original model.

### Description of evaluation datasets used to estimate continuous RIs

To showcase the application of the developed pipeline, we evaluate pediatric datasets of three biomarkers that are known for their extensive dynamics during physiological development: Alkaline phosphatase (ALP), Creatinine (CREA), and Hemoglobin (HB). We retrieved pseudonymized test results for boys and girls with ages 0–18 years measured during routine care at a tertiary care center (Department of Pediatrics and Adolescent Medicine, University Hospital Erlangen, Germany). The measurements were performed on Roche cobas^®^ instruments (ALP, CREA) and SYSMEX instruments (HB) between 2010 and 2022 (Table 1). The datasets contained the numeric test results, sex of the subject, the age in days, and a non-traceable subject identifier. Both in- and outpatient test results were included in the datasets and no outlier exclusion was performed prior to applying the pipeline. Use of pseudonymized patient datasets obtained during patient care without patients’ explicit consent is in accordance with the applicable German/Bavarian regulations. The performed analyses of pediatric datasets have been approved and the need for informed consent was waived by the Ethical Review Boards of the University Hospital Erlangen, reference numbers 97_17 Bc and 216_21 Bc.

**Table 1:** Description of input datasets.

Analyte	Number of samples		Number of subjects		Unit	Instrument
	Male	Female	Male	Female		
ALP	78,535	61,933	18,645	16,786	U/L	Roche cobas INTEGRA 800/cobas c501
CREA	125,572	99,036	21,507	18,655	mg/dL	Roche cobas INTEGRA 800/cobas c501
HB	164,687	131,336	27,155	24,232	g/dL	SYSMEX XE-2100

The table shows characteristics of the input datasets used for the application of the pipeline. The data was obtained from the Department of Pediatrics and Adolescent Medicine, University Hospital Erlangen, Germany. ALP, alkaline phosphatase; CREA, creatinine; HB, hemoglobin.

### Comparison to continuous and discrete RIs based on other methods

To evaluate the quality of results of the developed pipeline, we compare the results obtained to continuous and discrete RIs, previously estimated using other populations and methods.

For the comparison, we include RIs established as part of the CALIPER project (*CA*nadian *L*aboratory *I*nitiative in *PE*diatric *R*eference Intervals)^21,36^, the PEDREF study (Next-Generation Pediatric Reference Intervals)^13,14^, and extracted from package inserts for the different assays provided by the manufacturers^23–27^. For PEDREF and for CALIPER we received continuous RIs, while the package inserts only contain discrete, age-group specific RIs.

The CALIPER project has established age- and sex-specific RIs for children from birth to adulthood for over 170 biomarkers using population-based methods. For a subset of these, continuous RIs are reported as well^21,22,37,38^. However, due to a limited number of samples from healthy neonates and young children, and the extensive dynamics during these early stages of life, the first 6 or 12 months of life are excluded. For ALP, CREA, and HB, continuous RIs were generated using nonparametric quantile regression with penalized splines, and the resulting RIs (2.5th and 97.5th percentiles) are reported for each half year of life^21,22^. Additionally, for ALP and CREA, continuous RIs have recently been established using LMS-based models^38^. As our datasets for ALP and CREA were measured on Roche cobas devices, but the CALIPER RIs are based on samples measured on Abbott ARCHITECT c8000 instruments, we transferred the reference limits (RLs) to Roche devices using the previously established equation for discrete RLs^39^.

PEDREF is a multi-center, data-driven project utilizing data from laboratory information systems from 13 German university hospitals to establish pediatric RIs^13^. For ALP and CREA fractional polynomial functions are provided to compute the RIs for boys and girls using age as an input parameter^14^. For HB, the RIs were established using penalized splines and the specific values are provided for each day of life from 0 to 18 years^13^.

## Results

We applied the developed fully automated pipeline to pediatric routine measurements of three biomarkers for boys and girls. In total, the number of samples analyzed reached from 61,933 samples for ALP (Girls) to 164,687 samples for HB (Boys) (Table 1). Table 1 provides an overview of the most important characteristics of the datasets, e.g. the number of samples and patients per biomarker. Additionally, Supplementary Fig. S1 shows the sex-specific age distribution per biomarker, revealing an overrepresentation of samples in the first year and month of life for all analyzed biomarkers. This overrepresentation is more pronounced for HB and CREA than for ALP. An analysis of the average computation time can be found in the Supplemental Material.

**Figure 2:**
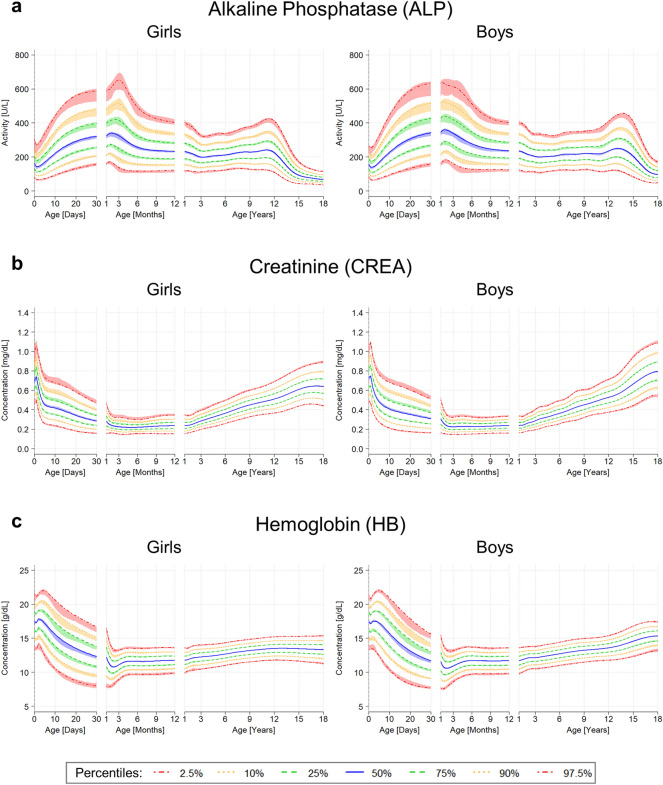
Estimated percentile charts with confidence intervals. Sex-specific percentile charts for alkaline phosphatase, hemoglobin, and creatinine, showing the 2.5th and 97.5th percentile (usually referred to as reference intervals) (dotted-dashed red lines), the 10th and 90th percentile (dotted yellow lines), the 25th and 75th percentile (dashed green lines) and the 50th percentile (solid blue line). The color-shaded area around the curves show the estimated 95% confidence interval potentially extended to include the estimates of the original model. The scale of the x-axis is threefold, showing the first 30 days of life, months from 1 to 12 and years from 1 to 18 to account for the dynamics in neonates and infants. For results shown on linear age scale, see Supplemental Fig. S2.

Applying the presented pipeline to these biomarker datasets enabled the generation of continuous RIs and percentile charts with CIs, as shown in Fig. 2. These percentile charts allow to accurately capture the physiological changes occurring throughout childhood. For the three analyzed biomarkers, these changes with age were most pronounced in infancy, early childhood (< 1 year) and during puberty (Fig. 2).

For ALP, we can observe an increase of RIs after birth that reaches its peak between month 3 and 4 (Fig. 2a). Already during these early stages of life, we can detect a difference between boys and girls, with higher percentiles and a sharper increase and peak in boys. A second increase and peak is shown during the age of ~13–15 years for boys and around ~11–13 years for girls, coinciding with the respective onset of puberty. Again, the peak is more pronounced for boys than for girls (Fig. 2a).

For CREA, there is a rapid decrease of RIs after birth until the age of 4 months with a steady and slight increase afterwards until the age of 18 years. For boys, we can observe a second, steeper increase again coinciding with the onset of puberty at the age of ~13 years (Fig. 2b). Further, for CREA we can observe fluctuations between the age of 0 and 2 years for girls and between 0 and 10 years for boys.

The percentiles for HB show an increase on day 1 after birth followed by a rapid decrease until ~50 days of life. Following that, the percentiles slightly increase. For girls, the percentiles stay constant over the remaining course of time, while for boys there is a second increase at the age of ~13 years (Fig. 2c).

Looking at the estimated CIs, we can observe that these are narrow when the estimated RIs are almost constant over time or there is only a slight and steady increase or decrease, e.g. for HB and CREA after the first months. For more pronounced and rapid changes, like in the first 30 days of life for all three biomarkers or at the peak at 3 months for ALP, the estimated CIs are broader, revealing an increased uncertainty.

**Figure 3:**
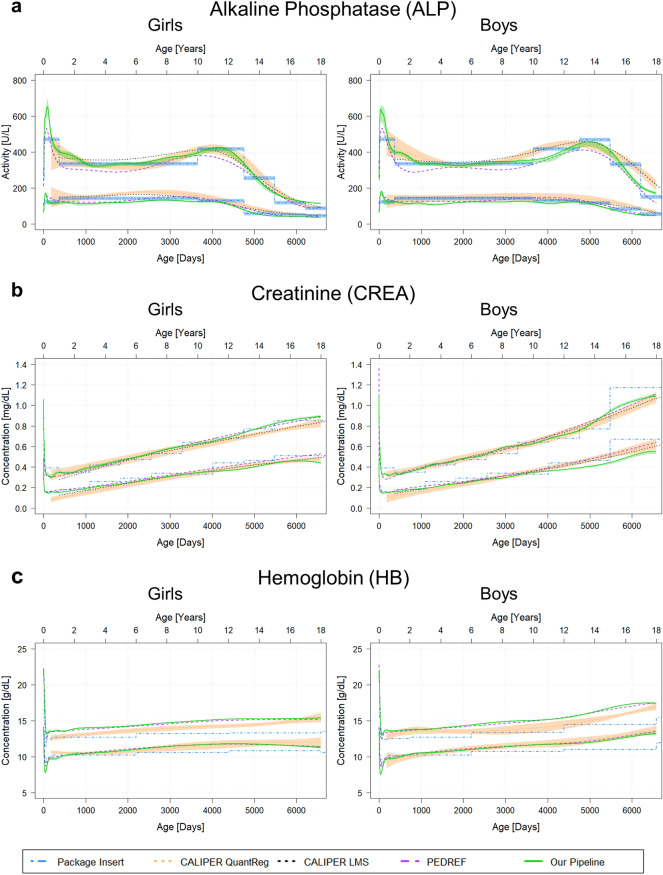
Comparison of estimated reference intervals to previously established discrete and continuous reference intervals. Each plot shows the estimated continuous reference intervals (RIs) (2.5th and 97.5th percentiles) with confidence intervals (CIs) (green solid lines and green shaded area) in comparison to the RIs (with CIs) established as part of the CALIPER study (dotted orange lines and orange colored region for results using quantile regression (QuantReg); dotted black lines for results using LMS method), the RIs determined within the PEDREF project (dashed purple lines) and the discrete RIs (with CIs) stated in the manufacturers’ package inserts (dashed-dotted blue lines and blue colored region).

In order to put our generated results into context, we compared the results to RIs established in other projects. Here, we included results from the CALIPER studies^21,22,38^, the multi-center PEDREF project^13,14^, as well as RIs stated in the package inserts provided by the manufacturers^23–27^. The comparisons are shown in Fig. 3.

Overall, we can observe a high agreement between the different methods for the analyzed biomarkers. All methods reveal the same trends in dynamics but also show some differences.

For ALP (Fig. 3a), the estimated continuous RI is overall highly similar across all methods for both girls and boys with some minor differences. For example, for girls, the lower RL reported by both CALIPER studies is higher between 1 and 12 years compared to results obtained by our pipeline and PEDREF. The upper RL for girls estimated by the CALIPER LMS approach is slightly higher compared to the other methods between 3 and 8 years. For the upper RL for boys, there is a slight difference in the second peak for boys revealing a broader peak and earlier onset in the CALIPER results and the package insert compared to the RL reported by PEDREF and our pipeline. For the first month and year, the upper RL estimated by the pipeline is slightly higher than the one established as part of the PEDREF study, for both girls and boys.

For CREA (Fig. 3b), again there is high agreement between our RIs and the ones generated as part of the PEDREF project and the CALIPER project. The same holds true for the discrete RIs stated in the package insert. Only for the lower RL for boys, we see slightly lower estimates reported by our pipeline for age greater than 11 years.

Looking at the results for HB (Fig. 3c), once again the RIs estimated by the presented pipeline are highly similar to the ones established as part of the PEDREF study. Comparing the results to the CALIPER study, we can observe that the lower RL is in high agreement as well. For the upper RL, CALIPER reports slightly lower values for girls until the age of ~16 years, as well as for boys between the age of ~4 and 18 years. After the first month until the age of ~5 years, the discrete lower RL is highly similar to the other methods, but then continues at a lower level. Regarding the upper RL, the values reported in the package insert are shifted towards lower values.

Altogether, we see a high concordance between the results of all five methods and studies. The discrete RIs reported in the method sheets show the same physiological trends as the continuous RIs but with reduced age-specific resolution.

## Discussion

Continuous RIs and percentile charts precisely capture the physiological dynamics that happen during the course of life^5,10,13^. Therefore, continuous RIs help facilitate and improve the interpretation of laboratory test results in medicine^7,9^. However, using population-based methods, ethical, practical and logistical challenges impede the establishment of such continuous RIs^5,11,40^.

Here, we provide a novel, data-mining approach that enables the automated estimation of such continuous RIs and percentile charts leveraging routine measurements. By using RWD, we circumvent the aforementioned challenges associated with direct methods. This idea of using routine measurements is also at the core of the PEDREF project. However, in contrast to previous approaches, where a sequential procedure using an indirect method and then applying manual smoothing with penalized splines or fractional polynomials was employed^13,14^, we developed an automated pipeline without any subjective user input or manual parameter tuning required. Thus, we provide the first parameter-less and fully automated approach to estimate continuous RIs. In addition, utilizing the distribution parameters estimated by the indirect method enables a weighted contribution of all raw data points, controlling the impact of pathological samples on the estimation of smooth curves. Employing the GAMLSS approach not only allows for the estimation of continuous RIs and percentile charts, but also provides a parametric model that can be used to compute any percentile for any arbitrary value of the covariate. Additionally, the pipeline facilitates the estimation of CIs using bootstrapping. Furthermore, we developed the pipeline in a modular way. Hence, in principle every other indirect method, which allows for the estimation of a parametric model of the non-pathological distribution, as well as any other algorithm that takes a weighted data set as input and estimates smooth curves can be employed.

Looking at the results generated by our pipeline and comparing them to continuous RIs from PEDREF and CALIPER, as well as to discrete RIs provided in the manufacturers’ package inserts, we see a high concordance between the different methods. Importantly, the overall dynamics and trends for the different biomarkers are conserved and highly similar over all methods and studies. Nevertheless, we observe some differences between the approaches, which can be caused by various reasons.

On the one hand, the underlying populations differ between the various methods. While CALIPER is based on a Canadian population, PEDREF and the datasets analyzed here use a German pediatric population, with PEDREF comprising data from 13 centers scattered throughout Germany and our datasets originating only from one tertiary care center in Southern Germany. Additionally, CALIPER and the results stated in the package inserts are based on apparently healthy children and direct methods, while the RIs from PEDREF and our pipeline are based on RWD and indirect methods. For the latter, different data pre-filtering methods can influence the estimated results^30,31,41^. In addition, the measurement devices can have an impact on the results^42^. The continuous RIs provided by the CALIPER project for ALP and CREA are based on Abbott ARCHITECT c8000 analyzers^21^ but transferred to Roche cobas devices, while the samples used in PEDREF were analyzed on instruments from different manufacturers (e.g. Roche, Ortho, Beckman Coulter, and Siemens)^14^. The data used in our analysis was measured on Roche cobas^®^ INTEGRA 800 and cobas^®^ c501 instruments. For HB, the data for all projects were measured on SYSMEX devices^13,22^.

On the other hand, the statistical methods utilized to estimate continuous RIs differ. For CALIPER, either quantile regression or the LMS method combined with sample collection studies was used^21,38^. In the PEDREF project, RIs were established using an indirect method with penalized splines or fractional polynomial functions in combination with manual tuning of smoothing parameters^13,14^. The developed pipeline, also based on RWD and an indirect method, integrates the results of the indirect method when computing continuous RIs using a sophisticated statistical approach, GAMLSS. Further, the applied smoothing strength, a highly subjective tuning parameter, differs between the methods. While this parameter is manually adapted for the PEDREF RIs and the CALIPER RIs, the developed pipeline automatically and objectively optimizes the smoothing strength.

Using the GAMLSS approach results not only in continuous RIs and percentile charts but also yields a parametric model. This model allows for future integration of results into laboratory information systems and estimation of any percentile and thus RL for any arbitrary age. Additionally, such a parametric model enables the transformation of test results into z-scores^13,14^.

Putting the results generated by our developed pipeline into medical context, we can see that the course of the estimated curves are in line with results that were previously established^16,21,43^. The estimated percentile charts reveal important differences during the physiological development between boys and girls, e.g. during puberty^16,43^ and capture the dynamics that occur throughout life with a high temporal resolution. Especially, in the first days and month this is difficult to achieve with a direct study, hence CALIPER does not report continuous RLs for the first half year or year of life. Discrete RIs are able to show the rough dynamics through childhood development but sometimes result in strong discontinuities between neighboring groups (e.g. Fig. 3a (ALP neonatal period)) or even gaps (e.g. Fig. 3b CREA 15 days of life until 2 months).

Continuous RIs and percentile charts enable to precisely capture the physiological dynamics that happen throughout life and thus facilitate the interpretation of test results. Furthermore, collection of longitudinal data for a patient in combination with age-dependent percentile charts may help to detect pathological changes earlier^13^. Due to the inclusion of age-dependent dynamics, these pathological changes can be better distinguished from any changes caused by physiological development.

## Limitations

The presented pipeline enables the automated estimation of percentile charts and a parametric model depending on the covariate age. The comparison to continuous or discrete RIs determined by other methods has shown a high agreement between the different approaches. Nevertheless, there are also some differences observable. As the underlying truth is unknown, we can just qualitatively evaluate the similarity of the methods. Thus, there is the need for a quantitative performance evaluation and comparison of the different approaches using simulated data. While this is available for the one-dimensional indirect methods^30^, a similar approach would also be highly beneficial for the estimation of continuous RIs. Such a simulation study would also help in answering important questions for the application of the pipeline, like the minimum number of samples required for the one-dimensional refineR estimation in each age partition.

In addition to such a simulation study, future work should also consider a rigorous clinical validation. While this manuscript describes the computational pipeline and shows the feasibility of its application, a clinical validation would ideally include a comparison of the proposed pipeline to a direct approach. The direct approach would operate on the same input dataset applying inclusion and exclusion criteria to obtain ‘healthy’ subjects, ensuring maximum comparability. Hence, access to patient data including clinical information on subjects and the definition of inclusion and exclusion criteria for each analyzed biomarker would be required. If certain clinical information is available, data prefiltering can improve the results of the indirect methods, e.g. only include measurements from samples that were taken in the morning times to account for the within-day variation of certain biomarkers^44^.

The pipeline also enables the computation of CIs for the percentile charts and continuous RIs using bootstrapping. While the bootstrap-based CIs provide an estimate of the underlying uncertainty, they are computationally inefficient and future work may focus on alternative approaches, e.g. analytical methods for CI estimation.

While we have shown that the pipeline already provides precise estimations of continuous RIs using routine measurements for pediatric populations with age in days as covariable, the computational efficiency of the approach can be improved, e.g. by enhancing the definition of discrete age groups depending on the dynamics of the input dataset.

Further, the current approach operates in a sequential procedure propagating the error made by the initial estimation of the indirect method further. This can for example be seen by the fluctuations observable for CREA (see Suppl. Fig. S3 for comparison between raw refineR estimation and smooth curves). Thus, to improve the overall estimation, future work may focus on developing an iterative procedure that is able to correct errors made in the initial estimation and thereby yield more robust results.

## Conclusion

The presented pipeline enables the automated generation of high-precision percentile charts and continuous RIs using routine measurements in combination with an established indirect method for RI estimation, refineR, and a sophisticated statistical approach to generate smooth curves, GAMLSS, thereby overcoming ethical and practical challenges. Continuous RIs and percentile charts capture the pronounced age-dependent dynamics and thus facilitate the interpretation of test results, ultimately improving patient care.

### Supplementary Information

Supplementary Information 1.

Supplementary Information 2.

## Data Availability

All components of the pipeline are available as open-source R-packages. The R code/scripts for executing the pipeline are included as Supplementary Material to this published article. The analyzed datasets are not publicly available but access can be granted upon reasonable request to the corresponding author, after approval by the data protection officer of the University Hospital Erlangen.
