# Supplementary material for: A pipeline for the fully automated estimation of continuous reference intervals using real-world data
Source: Sci Rep. 2023 Aug 18;13:13440. doi: 10.1038/s41598-023-40561-3 (PMC10439150; doi:10.1038/s41598-023-40561-3)
Supplement: Supplementary file 1 — Supplementary Information 1. [file 41598_2023_40561_MOESM1_ESM.docx]

# **A Pipeline for the Fully Automated Estimation of Continuous Reference Intervals using Real-World Data**

# **Author list:**

Tatjana Ammer^1, 2^, André Schützenmeister^2^, Hans-Ulrich Prokosch^1^, Manfred Rauh^3^, Christopher M Rank^2+^ and Jakob Zierk^3, 4+,*^

^+^These authors contributed equally to the publication.

# ***Corresponding author:** Jakob Zierk, [jakob.zierk@uk-erlangen.de](mailto:jakob.zierk@uk-erlangen.de)

# **Affiliations:**

^1^Friedrich-Alexander-Universität Erlangen-Nürnberg, Chair of Medical Informatics, Erlangen, Germany

^2^Roche Diagnostics GmbH, Penzberg, Germany

^3^Universitätsklinikum Erlangen, Department of Pediatrics and Adolescent Medicine, Erlangen, Germany

^4^Universitätsklinikum Erlangen, Center of Medical Information and Communication Technology, Erlangen, Germany


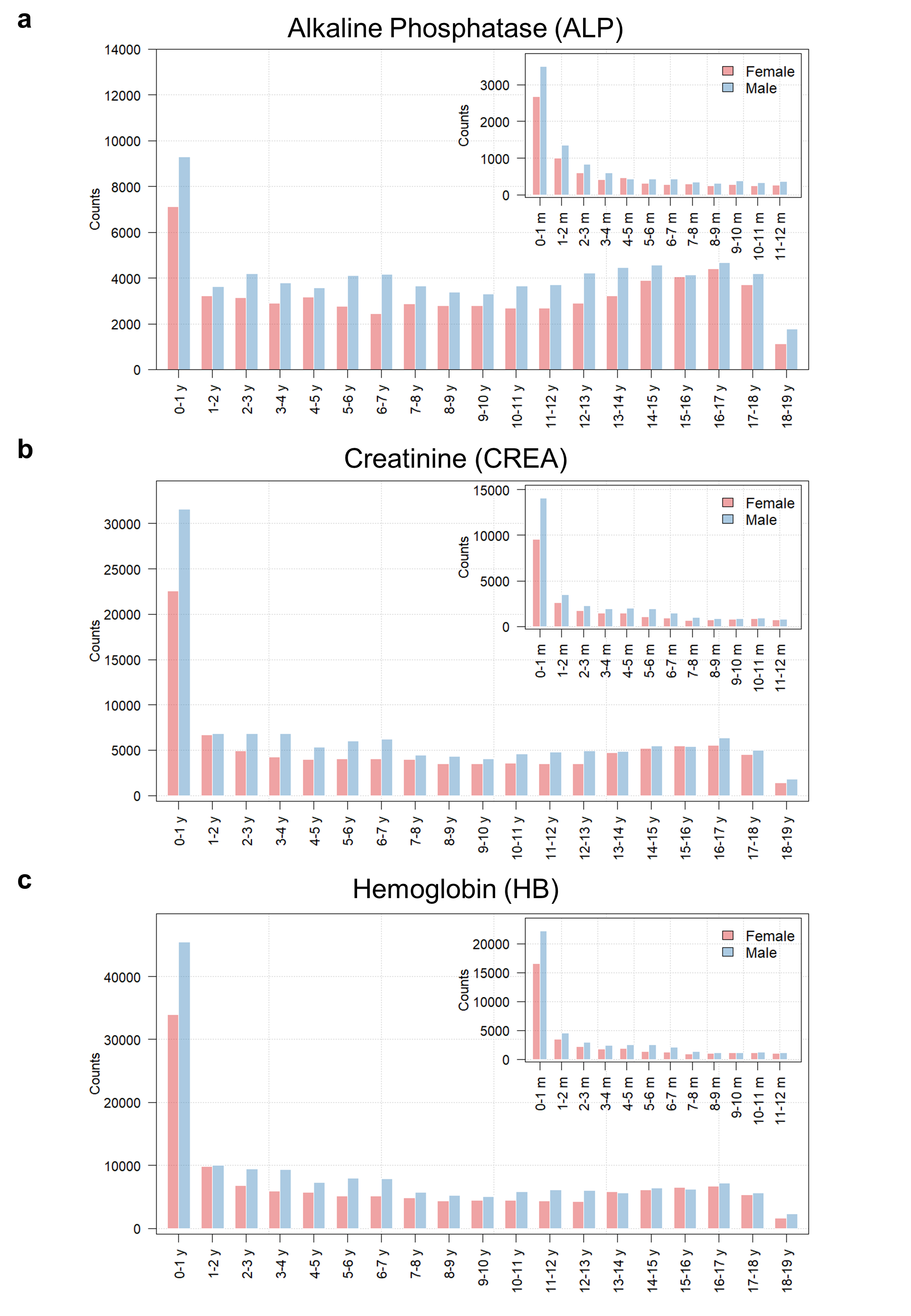


**Supplemental Figure S1. Sex- and age-specific distributions of samples.** The plots show the age distribution of the samples in the input datasets in years (y) for male (blue) and female (pink) for the three different biomarkers (alkaline phosphatase, hemoglobin, and creatinine). The inset plot shows the distribution for the first year of life in more detail (in months (m)).

**Runtime analysis**

The pipeline was executed on an Intel Core i9-9820X processor with 10 physical cores. The estimation of the original model on the whole data set (i.e. one run of the pipeline) takes about ~3 hours in single-core configuration. Computing confidence intervals with 100 bootstrap iterations adds up to ~19 hours in multi-core configuration.


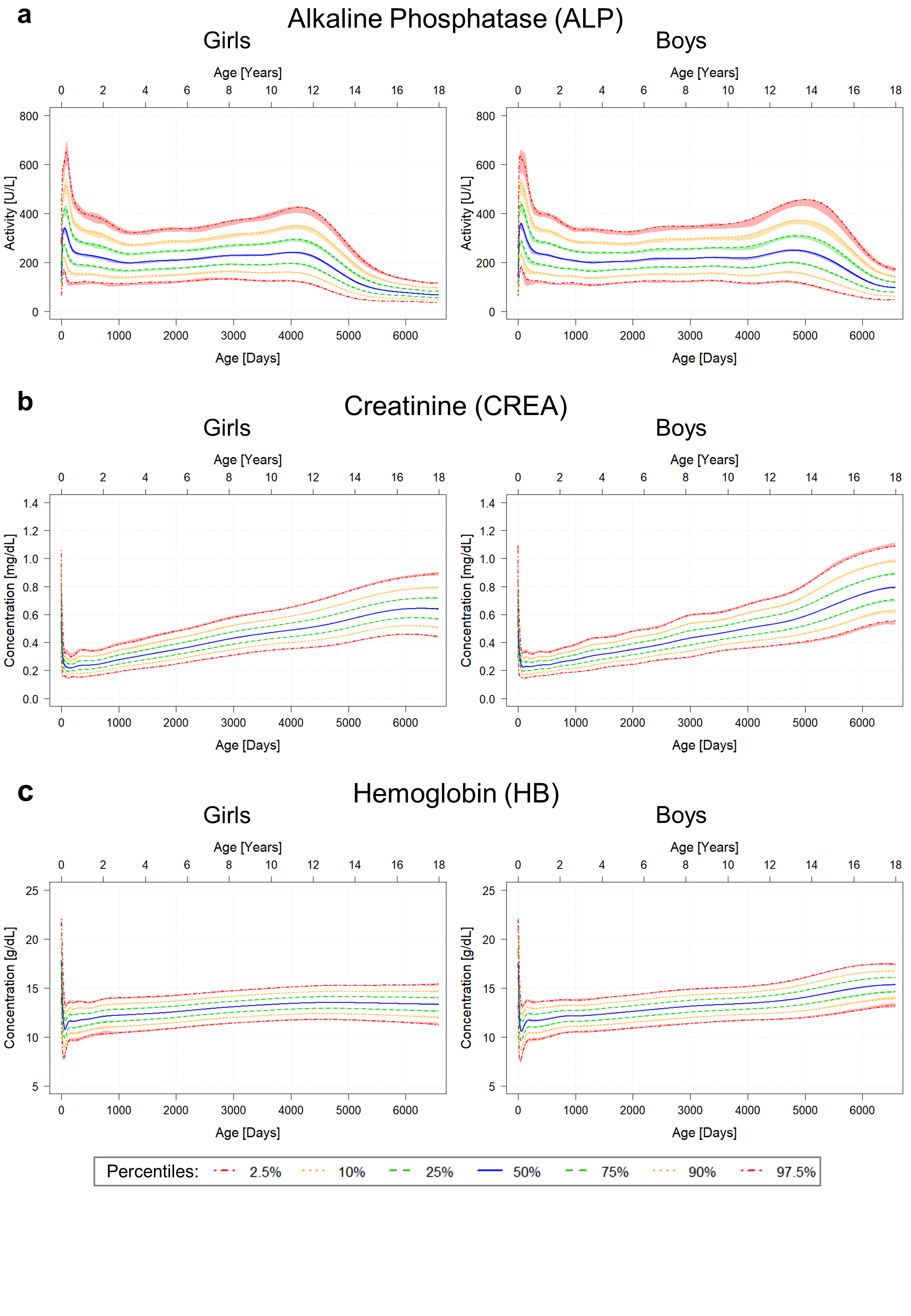


**Supplemental Figure S2: Estimated percentile charts with confidence intervals.** Sex-specific percentile charts for alkaline phosphatase, hemoglobin and creatinine, showing the 2.5^th^ and 97.5^th^ percentile (usually referred to as reference intervals) (dotted-dashed red lines), the 10^th^ and 90^th^ percentile (dotted yellow lines), the 25^th^ and 75^th^ percentile (dashed green lines) and the 50^th^ percentile (solid blue line). The color-shaded area around the curves show the estimated 95% confidence interval (CI) potentially extended to include the estimates of the original model. For results shown with alternative scaling of age, see Fig. 2.


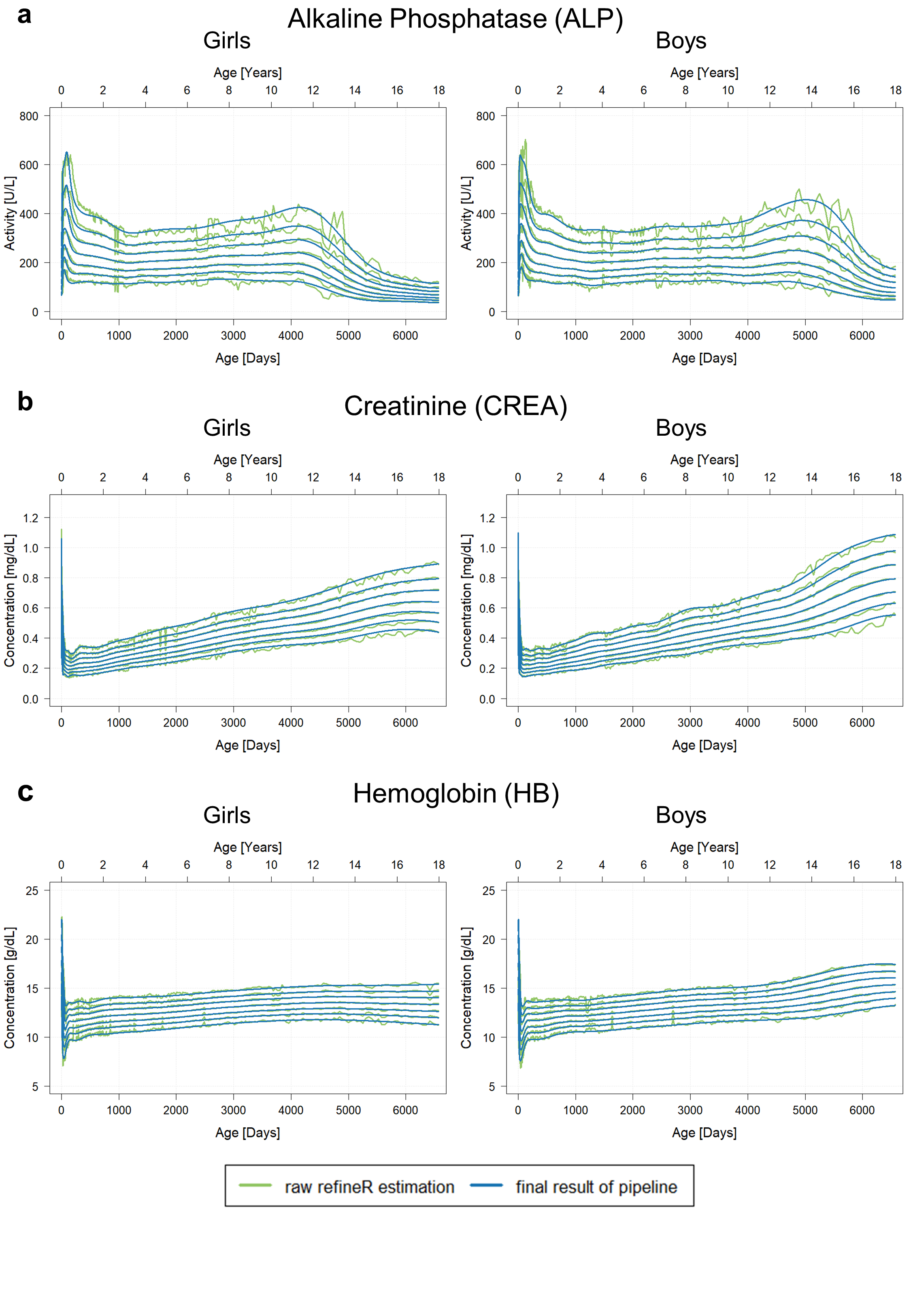


**Supplemental Figure S3: Comparison of raw refineR estimations and smooth final curves.** Sex-specific percentile charts for alkaline phosphatase, hemoglobin and creatinine, showing the 2.5^th^, 10^th^, 25^th^, 50^th^, 75^th^, 90^th^, and 97.5^th^ percentile for the refineR estimation (green) and the smooth final results of the pipeline (blue).
